# Overcoming immunotherapy resistance in breast cancer: a novel strategy by targeting the integrated stress response

**DOI:** 10.3389/fcell.2026.1720480

**Published:** 2026-03-19

**Authors:** Jian Yue, Tianyi Pu, Dele He, Yangyong Luo, Simin Ruan, Huahan Li, Jianwei Zhan, Guosen Su, Jianyuan Su, Sheng Chen, Guoxing Huang

**Affiliations:** 1 Department of Breast Surgery, Gaozhou People’s Hospital, Gaozhou, China; 2 Department of Pathology, Chongqing Hospital of Jiangsu Province Hospital, Chongqing, China; 3 Department of Neurosurgery, Gaozhou Hospital of Traditional Chinese Medicine, Gaozhou, China; 4 Department of Emergency Medicine, Taishan People’s Hospital, Taishan, China

**Keywords:** breast cancer, eIF2α-ATF4 axis, immune checkpoint inhibitors, immunotherapy resistance, integrated stress response (ISR), tumor microenvironment

## Abstract

Immunotherapy resistance remains a major obstacle in treating breast cancer, particularly aggressive subtypes like triple-negative breast cancer (TNBC). This review delineates the pivotal role of the Integrated Stress Response (ISR) as a central metabolic-immune regulator driving this resistance. The ISR is activated in the tumor microenvironment (TME) by diverse stressors—including hypoxia, nutrient scarcity, and ER stress—via four upstream kinases (PERK (PKR-like ER kinase), GCN2, PKR, HRI). These kinases converge to phosphorylate eukaryotic initiation factor 2α (eIF2α), leading to the selective translation and robust activation of the transcription factor ATF4. The ensuing ATF4-driven program fosters an immunosuppressive TME through multifaceted mechanisms: tumor-intrinsic upregulation of PD-L1, secretion of immunosuppressive exosomes, metabolic reprogramming that depletes critical amino acids, and direct impairment of T cell function and antigen presentation. Concurrently, ISR activation in immune cells—such as myeloid-derived suppressor cells (MDSCs) and dendritic cells—further dampens antitumor immunity. Targeting the ISR with small-molecule inhibitors (PERK or GCN2 inhibitors, ISRIB) or repurposed agents (metformin) demonstrates compelling preclinical efficacy in reversing immunosuppression and synergizing with immune checkpoint inhibitors. Biomarker-driven strategies, including ISR gene signatures and p-eIF2α immunohistochemistry, offer promising avenues for patient stratification. Thus, pharmacological targeting of the ISR represents a strategically viable approach to reprogram the immunosuppressive TME and overcome immunotherapy resistance in breast cancer, warranting urgent clinical investigation.

## 1 Introduction

Of the various malignancies affecting women globally, breast cancer continues to be a leading cause of cancer-associated mortality (Bray et al., 2024). While immunotherapeutic approaches—especially immune checkpoint blockade—have transformed oncology practice, their clinical benefit in breast cancer, notably the triple-negative subtype, remains constrained by both intrinsic and adaptive resistance (Liu Y. et al., 2023). These therapeutic shortcomings underscore the need to identify novel targets capable of favorably reshaping the tumor immune microenvironment and augmenting response to immunotherapy.

The integrated stress response (ISR) constitutes an evolutionarily conserved signaling network that coordinates cellular adaptation to diverse extrinsic and intrinsic challenges, such as nutrient deficiency, hypoxia, oxidative stress, and proteotoxic burden (Malnassy et al., 2024). Central to this pathway are four upstream kinases—PERK (Calvo et al., 2023), GCN2 (Rajanala et al., 2019), PKR (Wong et al., 2025), and HRI (Tian et al., 2024)—that collectively phosphorylate the eukaryotic initiation factor 2α (eIF2α) (Tauber and Parker, 2019). This post-translational modification attenuates global cap-dependent protein synthesis while facilitating the preferential translation of specific adaptive transcripts, most notably activating transcription factor 4 (ATF4) (He et al., 2023). Through ATF4-mediated transcriptional regulation, the ISR modulates a spectrum of biological processes spanning amino acid metabolism, redox homeostasis, autophagy, and ferroptosis (He et al., 2023).

### 1.1 Scope note

**FIGURE 1:**
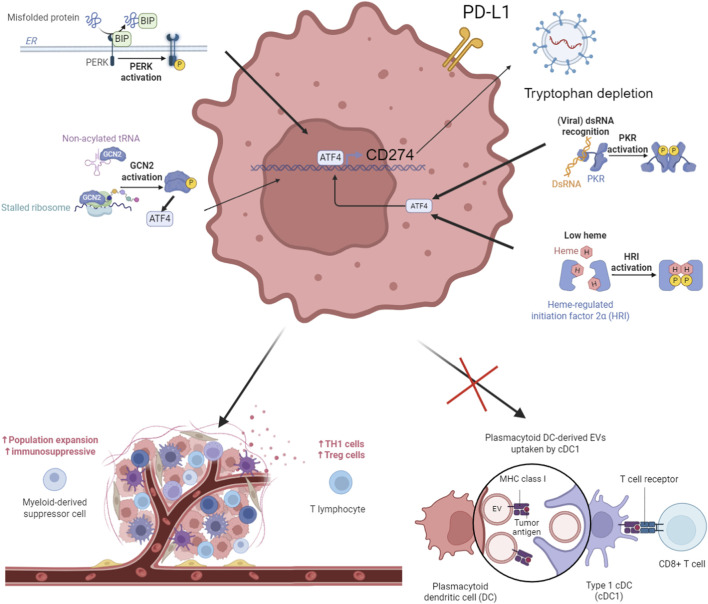
Schematic diagram of the ISR-mediated immunosuppressive mechanism in breast cancer. Created with BioRender.com. OB29GZF1RR.

Here we use “ISR” to refer primarily to the eIF2α–ATF4-centered ISR core (including context-dependent CHOP/GADD34 outputs). Other stress pathways (IRE1α/XBP1) are discussed only where they mechanistically intersect with the ISR core and influence immune phenotypes in breast cancer. A schematic overview of ISR-mediated immunosuppressive mechanisms in breast cancer is shown in Figure 1.

Accumulating studies reveal that the ISR not only supports cell-autonomous survival but also actively shapes intercellular crosstalk within the tumor microenvironment (Mijit et al., 2023). In breast cancer, persistent ISR activation has been associated with disease progression (Yeh et al., 2025), metabolic rewiring, and induction of immunosuppression. Critically, ISR-mediated adaptations in both malignant and immune cells (Thomas-Jardin et al., 2025) contribute to establishing an immune-evasive niche that undermines the efficacy of immunotherapeutic interventions.

A key conceptual distinction is that acute, adaptive ISR activation can transiently restore proteostasis and promote survival, whereas sustained or repetitive ISR activation may become maladaptive, reprogramming metabolism and immune interactions in ways that favor immune evasion. Importantly, the same ISR nodes may exert opposite immune consequences depending on timing, amplitude, and resolution capacity. In practical studies, this distinction can be operationalized by tracking (i) the dynamics of p-eIF2α and ATF4 (rapid induction followed by resolution versus persistently elevated signaling), and (ii) the balance of ISR “resolution” versus “pro-apoptotic” outputs, for example, GADD34/PPP1R15A (feedback dephosphorylation/resolution) relative to CHOP/DDIT3 (stress escalation). These markers can guide whether ISR modulation is more likely to restore anti-tumor immunity or inadvertently blunt beneficial stress-associated immunogenicity.

This review advances the perspective that the ISR acts as a critical metabolic-immune interface in breast cancer, exerting dual functions that support tumor survival while simultaneously impairing antitumor immunity. The ISR serves as a central hub that senses metabolic stresses in the tumor microenvironment—such as nutrient scarcity and hypoxia—and translates these signals into coordinated immunosuppressive outputs. This interface function is pivotal because it concurrently regulates both the adaptive survival mechanisms of tumor cells and the functional states of immune cells, representing a fundamental mechanism underlying immunotherapy resistance. To address the challenge of immunotherapy resistance, we propose the strategic targeting of specific ISR components as a means to disrupt this immunosuppressive network. Such an approach holds promise for re-sensitizing breast tumors to immune-mediated destruction and may offer a viable combinatorial strategy for improving therapeutic outcomes.

## 2 Molecular mechanisms of ISR in breast cancer

For each mechanism linking ISR signaling to immune phenotypes, we annotate the dominant level of supporting evidence as: (i) correlative (human patient datasets or associations), (ii) mechanistic (*in vitro* or *ex vivo* functional experiments), (iii) causal (*in vivo* genetic perturbation or well-controlled functional studies demonstrating necessity/sufficiency), and (iv) pharmacologic reversibility (rescue/phenotype reversal using ISR-targeting agents or pathway-selective inhibitors).

### 2.1 Activation mechanisms of ISR kinases in breast cancer

In breast cancer, the ISR is centrally coordinated through the phosphorylation of eIF2α, a key regulatory event catalyzed by four upstream kinases—PERK, GCN2, PKR, and HRI. These kinases are selectively activated by distinct stressors prevalent within the tumor microenvironment (TME), ultimately leading to broad suppression of protein synthesis alongside the selective upregulation of transcripts essential for cellular adaptation (Yadav et al., 2017).

PERK is predominantly activated by endoplasmic reticulum (ER) stress, a condition frequently induced by hypoxia (Yan et al., 2022), nutrient deprivation (Marjon et al., 2004), reactive oxygen species (ROS) (Sassano et al., 2025), and high protein-folding demand. Activation involves its dissociation from the chaperone BiP/GRP78, followed by dimerization and autophosphorylation, which enables eIF2α phosphorylation (Bambang et al., 2009). PERK can also be stimulated by lipid saturation within the ER membrane, highlighting a connection between metabolic imbalance and ISR induction. In triple-negative breast cancer (TNBC), persistent PERK activation is associated with aggressive tumor behavior and diminished treatment response (Wang H. et al., 2022). Beyond ISR components, other molecular features such as OBFC2A have also been identified as potential prognostic markers in TNBC, underscoring the complexity of this aggressive subtype (Wu et al., 2021).

GCN2 responds to amino acid deficiency—common in poorly perfused tumors—by detecting uncharged tRNAs (Yuan et al., 2017). In TNBC, metabolic stress triggers the GCN2/eIF2α/ATF4 axis to upregulate the long non-coding RNA UBA6-AS1, which enhances PARP1 activity and supports cell survival, potentially influencing PARP inhibitor sensitivity (Wu Y. Z. et al., 2022). Furthermore, inhibiting GCN2 disrupts asparagine synthesis, suppresses protein translation, and activates stress-related MAPK signaling, thereby sensitizing cancer cells with low basal ASNS levels to l-asparaginase and promoting apoptosis (Nakamura et al., 2018).

PKR is activated by double-stranded RNA (dsRNA), which may originate from viral elements, endogenous retroviruses, or damaged cellular RNA in the TME (Mitchell et al., 2017; Cuddihy et al., 1999). In TNBC, UBR5 upregulates PKR, which subsequently activates the STAT1–IRF1 pathway to promote IFN-γ-induced PD-L1 expression, contributing to immune evasion (Zhang et al., 2020).

HRI, known for its role in erythroid adaptation to iron deficiency, phosphorylates eIF2α to inhibit general translation while promoting an ATF4-driven adaptive program that preserves mitochondrial function during erythropoiesis (Zhang et al., 2019). In breast cancer, HRI mediates translational suppression under nitric oxide exposure and, together with PKR, induces pronounced cytostasis under severe oxidative stress—a response attenuated in normal epithelial cells by the endogenous PKR inhibitor p58 (Pervin et al., 2008).

Beyond these canonical pathways, the TME imposes context-specific stresses that modulate ISR kinase activity in a cell-type-dependent manner. Single-cell RNA sequencing reveals heterogeneous expression patterns of ISR kinases across tumor subpopulations (Verginadis et al., 2022). For example, methionine deprivation activates the ISR in TNBC through a mechanism independent of GCN2 and PERK (Rajanala et al., 2019), indicating that nutrient competition within the TME can trigger non-autonomous kinase activation. Functional redundancy and complementarity among kinases are also emerging as important themes. The traditional formulation Tong-Xie-Yao-Fang ameliorates irritable bowel syndrome by suppressing intestinal apoptosis via the GCN2/PERK–eIF2α–ATF4 cascade (Zhang et al., 2022). Additionally, HRI’s role is expanding beyond iron homeostasis; it is also activated by chemotherapy-induced oxidative stress, such as from doxorubicin, facilitating survival and therapy resistance.

The activation landscape of the ISR varies significantly across breast cancer molecular subtypes. Integrated multi-omics studies have further delineated the distinct molecular features of subtypes such as high-grade ER + HER2-breast cancer, highlighting the importance of subtype-specific context for understanding stress response pathways (Wang K. et al., 2022). Basal-like/TNBC, characterized by high genomic instability, rapid proliferation, and substantial metabolic stress, typically exhibit stronger basal activation of the ISR, particularly through the PERK and GCN2 pathways. In contrast, HER2-positive or Luminal subtypes may be more susceptible to ISR activation by specific insults, such as the ER stress induced by certain targeted therapies (Chen et al., 2020; Li X. et al., 2022). Furthermore, functional redundancy among the four upstream kinases (PERK, GCN2, PKR, HRI) adds another layer of complexity. Single-cell RNA sequencing revealed the heterogeneous expression of these ISR genes among transitional cells, enabling compensatory activation of one pathway component when another was inhibited to maintain overall ISR signaling (Han et al., 2023). This subtype-specificity and network-level buffering suggest that monotherapy targeting a single kinase may have limited efficacy, underscoring the need for strategies that simultaneously target multiple nodes or the central downstream effector ATF4 for a more effective and personalized therapeutic approach.

### 2.2 Downstream effects of ISR activation

Phosphorylation of eIF2α induces a rapid reprogramming of gene expression, largely orchestrated by the transcription factor ATF4 (Zielke et al., 2021). For instance, ATF4 mediates loperamide-induced reticulophagy and autophagic cell death in glioblastoma through upregulation of ER stress and reticulophagy receptors RETREG1 and TEX264 (Zielke et al., 2021).

ATF4 coordinates a broad transcriptional network regulating multiple adaptive processes. It upregulates genes involved in amino acid metabolism (Zhao et al., 2016)- such as ASNS (Zou Y. et al., 2024), SLC7A11/xCT (He et al., 2023), and SLC1A5 (Zhou et al., 2024) —to maintain amino acid availability. Furthermore, ISR-driven metabolic reprogramming can alter key metabolite pools, thereby influencing the epigenetic landscape. For instance, ATF4 mediates the transcriptional activation of the Ddit4 gene, which is induced by DNA hypomethylation resulting from a disrupted methionine cycle, thereby driving skeletal muscle atrophy in cancer cachexia (Lin et al., 2025). It also enhances antioxidant defense via NRF2 and its target genes (Sarcinelli et al., 2020), and exerts dual control over cell survival by modulating both pro-apoptotic CHOP and anti-apoptotic BCL-2 family members (Rozpedek et al., 2016). The activity of the ISR pathway is itself subject to fine-tuning at the epitranscriptomic level. For example, the m6A methyltransferase METTL16 can enhance the stability of ATF4 mRNA through m6A modification, thereby amplifying ISR signal output and suppressing ferroptosis to promote cholangiocarcinoma progression (Zhao et al., 2025). This indicates that targeting RNA modifications could emerge as a novel strategy for intervening in the ISR pathway (Zhao et al., 2025). In immune regulation, ATF4 activation through the PERK/eIF2α pathway upon STING loss induces CHOP-dependent, GSDME-mediated pyroptosis, boosting antitumor immunity in renal carcinoma (Wu et al., 2024).

The metabolic adaptations mediated by the ISR also significantly influence non-apoptotic cell death pathways. On one hand, ATF4 upregulates SLC7A11 to promote glutathione synthesis, thereby suppressing ferroptosis (He et al., 2023). On the other hand, under specific contexts such as the loss of STING signaling, the ISR can induce GSDME-mediated pyroptosis via the ATF4-CHOP axis (Li X. et al., 2021). Consequently, the activation status of the ISR is a critical determinant of breast cancer cell susceptibility to metabolism stress-induced ferroptosis and pyroptosis.

Global translational repression resulting from eIF2α phosphorylation reduces the synthesis of most proteins, including tumor antigens, thereby impairing antigen presentation and immune surveillance. In CD8^+^ T cells, GCN2-mediated eIF2α phosphorylation is critical for sustaining T-cell receptor signaling and preventing tryptophan starvation-induced necrosis in glioblastoma (Rashidi et al., 2020).

The ISR also contributes significantly to therapy resistance in breast cancer. PERK–ATF4 signaling promotes protective autophagy (Vanhoutte et al., 2021), whereas PRMT1-mediated methylation of ATF4 attenuates this pathway, reducing doxorubicin-induced ER stress and cardiotoxicity (Kim et al., 2022).

Crosstalk between the ISR and oncogenic pathways further drives tumor progression. For example, the ISR interfaces with key survival and inflammatory pathways such as NF-κB, which itself is regulated by specific proteins like Epsin 3 to influence apoptosis in breast cancer (Wu Q. et al., 2022). This interplay adds another layer of complexity to the cellular stress adaptation network. In HER2-positive and TNBC subtypes, ISR components interact with MYC and HIF1α signaling. For example, the lncRNA-encoded protein ERSP facilitates nuclear translocation of XBP1s, amplifying the IRE1α/XBP1s arm of the unfolded protein response and accelerating TNBC progression (Chen et al., 2024).

TF4 also engages in metabolic-immunological crosstalk: under low arginine conditions, the ATF4–SLC7A11 axis promotes glutathione synthesis, driving T cell reprogramming into immunosuppressive Treg-like phenotypes (Liu T. et al., 2023). Similarly, the oncogene c-Myc upregulates ATF4 via GCN2 kinase, and the ATF4-4E-BP1 axis acts as a critical rheostat to alleviate proteotoxic stress and enable tumor progression by balancing MYC-driven protein synthesis (Tameire et al., 2019). Metabolomic studies further delineate the ISR’s role in shaping the metabolic landscape: Low arginine levels in the tumor microenvironment reprogram T cells into immunosuppressive Treg-like cells via the ATF4-SLC7A11-GSH axis (Zou Z. et al., 2024). Cell fate decisions under ISR activation are highly context-dependent. In hepatocellular carcinoma, the lncRNA GOLGA2P10, induced by the PERK/ATF4/CHOP cascade, enhances cell survival by upregulating BCL-xL and inactivating pro-apoptotic BAD via phosphorylation (Wu et al., 2020). The ISR also interfaces with non-apoptotic cell death mechanisms; CHOP promotes renal ischemia-reperfusion injury by activating caspase-3, which simultaneously induces apoptosis and GSDME-mediated pyroptosis (Ma et al., 2024).

## 3 ISR modulates the breast cancer immune microenvironment

The ISR and the related Unfolded Protein Response (UPR) are increasingly identified as pivotal modulators of the tumor immune landscape. PERK signaling, for instance, supports the survival of dormant disseminated tumor cells while simultaneously fostering an immunosuppressive niche through NK cell-mediated impairment of myeloid cell function (Calvo et al., 2023; Neo et al., 2024).

### 3.1 Tumor-intrinsic mechanisms

The expression of the immune checkpoint protein PD-L1 is intricately regulated by the ISR. Inhibition of PERK leads to eIF2α dephosphorylation, which in pancreatic β cells increases PD-L1 stability in a Golgi membrane protein 1-dependent manner, thereby diminishing immunogenicity and delaying autoimmune diabetes (Yuan et al., 2021). Conversely, Liensinine-induced ER stress activates ATF4, which suppresses HIF-1α, resulting in PD-L1 downregulation, reinvigoration of CD8^+^ T cells, and alleviation of T cell exhaustion (Liu J. et al., 2025). Studies in cancer cells demonstrate that PERK, activated by the elevated ceramide levels induced by DTX-CPT-Gel therapy, mediates an unfolded protein response that promotes immunogenic cell death and ultimately clears metastatic tumors (Kar et al., 2023).

Beyond PD-L1, the immunomodulatory function of ATF4 may extend to other co-inhibitory immune checkpoints. ATF4 acts as a central mediator that translates chronic integrated stress in the TME into CD8^+^ T cell dysfunction and serves as a key barrier to effective immunotherapy (Alicea Pauneto et al., 2025). Although direct evidence in breast cancer remains to be fully established, ATF4, as a broad transcriptional regulator, has the potential to govern the expression of these molecules through direct or indirect mechanisms, thereby collectively constituting a multiple immune checkpoint barrier. This provides a theoretical rationale for combining ISR targeting with the blockade of multiple immune checkpoints.

Cellular stress influences intercellular communication via exosomes. For example, melatonin-loaded exosomes (Mel@M2-exos) alleviate ER stress and drive macrophage polarization from a pro-inflammatory M1 to an anti-inflammatory M2 state, which resolves periodontal inflammation (Cui Y. et al., 2023). Tumor-derived exosomes from breast cancer cells can carry immunosuppressive cargoes, such as PD-L1 and microRNAs, which directly impair T cell activation. For instance, metastatic breast cancer cells that are quiescent and exhibit a high ISR rely on PERK for survival, making them uniquely vulnerable to eradication by the PERK inhibitor HC4 (Calvo et al., 2023).

ISR activation orchestrates metabolic shifts that favor immune evasion. In TNBC, the GCN2/eIF2α/ATF4 axis is activated under metabolic stress, upregulating the long non-coding RNA UBA6-AS1 to enhance PARP1 activity and support cell survival, providing a direct link between ISR and metabolic adaptation specific to this aggressive subtype (Wu Y. Z. et al., 2022). Tryptophan scarcity activates GCN2, leading to upregulation of the SLC7A5 transporter. This enhances the uptake of kynurenine, a tryptophan metabolite, which potently activates the aryl hydrocarbon receptor (AHR) to drive Treg differentiation (Solvay et al., 2023). Furthermore, ISR-driven enhancement of serine and glycine metabolism supports nucleotide production and antioxidant defense, reinforcing an immunosuppressive tumor microenvironment.

The transcriptional control of PD-L1 by the ISR is complex. PD-L1 levels show correlation with IRF1, eIF2α, and ATF4, but not with STAT1/STAT2, indicating a specific connection between the PD-1/PD-L1 axis and the integrated stress response (Chang et al., 2018). The lncRNA UBA6-AS1, whose expression is induced by the GCN2/eIF2α/ATF4 cascade under metabolic stress, enhances PARP1 activity to support TNBC cell survival (Wu Y. Z. et al., 2022). The ISR also modulates the expression of other immune regulators, such as Thbs1, which activates PERK to drive ATF4-mediated autophagy, a pathway critically involved in controlling cardiomyocyte size (Vanhoutte et al., 2021).

The regulatory effects of the ISR on the immune microenvironment differ markedly among breast cancer subtypes. In TNBC, frequent genomic instability and metabolic stress lead to sustained activation of the ISR, particularly the PERK and GCN2 pathways, thereby driving upregulation of PD-L1, exosome-mediated immunosuppression, and Treg differentiation, ultimately establishing a highly immune-evasive microenvironment. In contrast, in HER2-positive breast cancer, targeted therapies such as trastuzumab can induce endoplasmic reticulum stress and activate PERK; however, the resulting immunosuppressive effects may be partially counterbalanced by the immune-activating properties of HER2-targeted therapy itself. Luminal breast cancers generally exhibit low basal ISR activity, but upon acquisition of endocrine therapy resistance, ATF4-mediated reprogramming of amino acid metabolism can emerge, promoting polarization of tumor-associated macrophages toward an M2 phenotype. These subtype-specific differences suggest that ISR-targeted therapeutic strategies should be individually tailored according to molecular subtype and treatment context (Chen et al., 2020; Li X. et al., 2022).

Targeting the ISR pathway has emerged as a novel strategy for modulating the tumor immune microenvironment. On the one hand, ISR activation can promote tumor cell–derived exosome secretion, such as PERK-mediated miR-27a-3p, thereby upregulating PD-L1 expression in macrophages and suppressing T-cell function. On the other hand, genetic ablation of GCN2 in myeloid cells markedly enhances CD8^+^ T-cell infiltration and improves their functional status by attenuating ATF4-mediated immunosuppressive programs (Halaby et al., 2019). Meanwhile, the ISR pathway can also directly induce ICD in tumor cells; for example, the cardiac glycoside oleandrin triggers ICD via activation of the PERK/eIF2α/ATF4/CHOP axis and synergizes with immune checkpoint inhibitors to enhance antitumor immunity (Li X. et al., 2021). Collectively, these findings indicate that interventions targeting the ISR pathway can bidirectionally modulate tumor immunity, providing new opportunities for combination strategies with existing immunotherapies (Yao et al., 2020).

### 3.2 Immune cell modulation

The ISR orchestrates an immunosuppressive tumor microenvironment by eliciting a spectrum of functional alterations across diverse immune cell populations. The following sections systematically detail the regulatory roles of the ISR in major immune cell types. These interconnected mechanisms collectively undermine antitumor immunity.

#### 3.2.1 T Cells

The ISR directly governs T cell function, persistence, and fate within the tumor microenvironment. Nutrient deprivation acts as a key trigger: GCN2 activation upon tryptophan scarcity phosphorylates eIF2α, which is essential not only for sustaining CD8^+^ T cell survival (Rashidi et al., 2020) but also for promoting their differentiation into an exhausted phenotype. More profoundly, low arginine conditions engage the ATF4–SLC7A11 axis, which drives glutathione synthesis and directly reprograms CD4^+^ T cells to acquire immunosuppressive, Treg-like functions (Zou Z. et al., 2024). This mechanism underscores how ISR-mediated metabolic stress can fundamentally reshape T cell fate decisions, forging a highly immunosuppressive milieu.

#### 3.2.2 Myeloid-derived suppressor cells (MDSCs)

The immunosuppressive capacity of MDSCs is enhanced by GCN2, which promotes ATF4 translation and fosters an immunosuppressive MDSC phenotype, thereby weakening the anti-tumor immune response (Halaby et al., 2019). Conversely, dabrafenib-induced GCN2 activation disrupts the development and function of polymorphonuclear MDSCs (PMN-MDSCs) by altering their transcriptional and metabolic states, ultimately reducing immunosuppression and enhancing anti-tumor immunity (Ciudad et al., 2024).

#### 3.2.3 Tumor-associated macrophages (TAMs)

PERK activation in TAMs reinforces an M2 immunosuppressive phenotype. This is achieved through ATF4-dependent upregulation of PSAT1, which enhances serine metabolism, mitochondrial respiration, and JMJD3-mediated epigenetic remodeling (Raines et al., 2022). Similarly, in macrophages, PSAT1 upregulation and the ensuing changes in serine metabolism can influence metabolites such as α-ketoglutarate, thereby regulating the activity of the histone demethylase JMJD3 to epigenetically reinforce M2 polarization (Raines et al., 2022). Separately, XBP1 in macrophages inhibits BNIP3-mediated mitophagy, leading to cytosolic mitochondrial DNA accumulation and activation of the cGAS/STING/NLRP3 inflammasome axis, which promotes liver fibrosis (Wang Q. et al., 2022).

#### 3.2.4 Dendritic cells (DCs)

XBP1 activation in tumor-associated DCs contributes to ovarian cancer progression by inducing pathological lipid accumulation, which impairs their ability to stimulate T cells. Deleting XBP1 in DCs restores their immunostimulatory function and improves survival (Cubillos-Ruiz et al., 2015).

#### 3.2.5 NKs

Although ISR–immunity studies in breast cancer have largely focused on T cells and myeloid populations, ISR-core programs may also influence NK-cell cytotoxicity, cytokine production, and persistence. Sustained eIF2α–ATF4 signaling could reshape metabolic fitness, stress tolerance, and the balance of activating versus inhibitory cues, thereby altering NK surveillance. However, breast-cancer–specific causal evidence remains limited, underscoring the need for compartment-resolved profiling and functional perturbation studies. Outstanding questions: (i) Which tumor-microenvironment stressors preferentially activate ISR signaling in NK cells versus tumor/stromal compartments? (ii) Does ISR modulation restore NK effector programs without compromising stress tolerance? (iii) How do ISR-targeting agents interact with NK-engaging modalities (ADCC-based antibodies or cytokine-based NK activation)?

#### 3.2.6 B cells

The ISR core may affect anti-tumor immunity not only through effector and myeloid states, but also by modulating antigen processing and presentation and potentially B-cell activation and antibody-mediated responses. Translational control downstream of eIF2α and ATF4-linked programs could influence peptide availability for MHC class I presentation, while chronic stress programs may shape antigen-presenting cell maturation and cross-presentation capacity. In breast cancer, these links remain underexplored and should be addressed to clarify whether ISR modulation primarily enhances immune visibility or, in certain contexts, reduces stress-associated immunogenicity. Outstanding questions: (i) Does ISR activation alter the antigenic peptide repertoire or MHC expression in ways that promote immune escape? (ii) How does ISR signaling in antigen-presenting cells impact priming versus tolerance? (iii) Are B-cell–rich immune-inflamed tumors associated with distinct ISR states that predict response to ISR-targeting combinations?

#### 3.2.7 Other immune cell types

The influence of the ISR extends to other immune contexts. In T-cell acute lymphoblastic leukemia (T-ALL), ATF4 upregulation via the GCN2-eIF2α pathway confers resistance to FGFR1 inhibitors by reprogramming amino acid metabolism—a vulnerability that can be targeted by combining FGFR1 and mTOR inhibition (Zhang et al., 2023). While the effects on B cells and neutrophils are less clear, the ISR may modulate antibody class-switching in B cells and polarize tumor-associated neutrophils (TANs) toward an N2 pro-tumor state. Furthermore, antibiotics like tedizolid can overcome venetoclax resistance in acute myeloid leukemia by inhibiting mitochondrial translation, thereby suppressing energy metabolism and triggering a lethal integrated stress response (Sharon et al., 2019). In breast cancer, a CXCL13-centered T cell gene signature predicts chemotherapy response, whereas stress-related metagenes show no consistent association (Stoll et al., 2014). Collectively, these interconnected mechanisms underscore how the ISR orchestrates a broad spectrum of immunosuppressive effects across diverse cell types within the TME, as summarized in Table 1.

**TABLE 1:** Mechanisms of ISR-driven immune suppression in breast cancer.

Target cell	Key ISR component	Immunosuppressive effect	Therapeutic intervention
Tumor cells	PERK-eIF2α-ATF4 axis	Increased PD-L1 transcription, leading to T cell exhaustion	PERK inhibitors (AMG-44)
	GCN2 sensing	Amino acid depletion, promoting Treg differentiation	GCN2 inhibitors (GCN2iB)
	Exosome secretion	Release of immunosuppressive factors (TGF-β, adenosine)	Exosome biogenesis blockers
T Cells	eIF2α phosphorylation	Increased expression of exhaustion markers (PD-1/TIM-3) and mitochondrial dysfunction	ISRIB (integrated stress response inhibitor B, eIF2B activator)
MDSCs	GCN2 activation	Enhanced amino acid catabolism, resulting in T cell suppression	Combined GCN2iB + anti-PD-1
Dendritic cells	ER stress (IRE1α/PKR)	Reduced MHC-I expression and impaired antigen presentation	NAC (ROS scavenger)

### 3.3 Therapeutic implications

Targeting the ISR presents a promising avenue for reversing immune suppression in breast cancer. Although PERK inhibition can block oleandrin-triggered immunogenic cell death (ICD) in breast cancer, its combination with immune checkpoint blockade synergistically activates dendritic cells and CD8^+^ T cells, leading to enhanced anti-tumor immunity, as validated in breast cancer mouse models (Li X. et al., 2021). Moreover, ISR-related gene expression profiles could serve as biomarkers to identify patients most likely to benefit from such combination therapies.

### 3.4 Outstanding questions

How do tissue-specific patterns of ISR activation affect immune cell recruitment and function across different breast cancer subtypes? Can modulating the ISR improve the efficacy of adoptive cell therapies or cancer vaccines? What is the impact of ISR inhibitors on the formation and function of tertiary lymphoid structures within breast tumors? The evolving comprehension of the crosstalk between stress response and immunity underscores the potential of targeting the ISR to break immunotherapy resistance in breast cancer.

## 4 Therapeutic targeting of ISR to enhance immunotherapy

### 4.1 Pharmacological inhibitors of the ISR

The ISR and the Unfolded Protein Response (UPR) are now established as critical drivers of immunosuppression within the tumor microenvironment. Consequently, pharmacologically targeting these pathways offers a viable strategy to counteract immune evasion and potentiate the effects of immunotherapeutic agents.

These agents disrupt key immunosuppressive mechanisms mediated by PERK activation in tumor cells and myeloid-derived suppressor cells (MDSCs). PERK inhibition (GSK2606414 (Mori et al., 2024)) has been shown to reduce PD-L1 expression, promote the infiltration of CD8^+^ T cells, and synergize with immune checkpoint blockade (Yao et al., 2020), demonstrating compelling efficacy in mouse models of breast cancer. Notably, next-generation PERK inhibitors like AMG-44 (Mijit et al., 2023) exhibit enhanced selectivity and an improved safety profile, mitigating the pancreatic toxicity associated with earlier compounds.

GCN2 acts as a sensor of amino acid scarcity, contributing to T cell dysfunction and enhancing MDSC activity. Pharmacological blockade of GCN2 with molecules such as GCN2iB (Yuan et al., 2022) reinstates T cell effector functions and alleviates immunosuppression in nutrient-deprived niches (Carlson et al., 2023), a finding recapitulated in co-culture systems using patient-derived organoids and autologous immune cells. This strategy shows particular promise in tumors reliant on asparagine, where combining GCN2iB with asparaginase therapy induces synergistic apoptotic cell death.

ISRIB functions by facilitating the assembly of the active eIF2B complex, thereby counteracting the translational shutdown enforced by eIF2α phosphorylation (Wu et al., 2025). This restoration of global protein synthesis improves antigen presentation and augments T cell activity. In patient-derived organoid models, ISRIB has demonstrated efficacy in enhancing chemotherapy response and represents a compelling candidate for combination with immunotherapy.

The clinical development of these inhibitors necessitates a thorough understanding of their mechanisms. For instance, the protein BZW1 drives pancreatic cancer progression and glycolysis under metabolic stress by activating the PERK/eIF2α axis to boost HIF1α and c-Myc translation, and its inhibition curbs tumor growth (Li Z. et al., 2022). Alternatively, inhibiting Ref-1 redox signaling induces the ISR, and coupling this with further ISR activation presents a novel therapeutic approach for pancreatic cancer (Mijit et al., 2023). The action of ISRIB extends to stabilizing the eIF2B decamer against destabilizing mutations, as seen in Vanishing White Matter Disease, thereby restoring its function and blocking the ISR (Wong et al., 2018). In breast cancer stem cells, reduced miR-183 expression elevates eIF2Bδ levels, which suppresses the ISR and facilitates metastasis and stemness (Gupta et al., 2023). Emerging strategies, such as proteolysis-targeting chimeras (PROTACs) against ISR components, promise more durable pathway inhibition and may circumvent resistance mechanisms seen with traditional inhibitors.

Moving beyond traditional small-molecule inhibitors, emerging therapeutic modalities offer novel tools for targeting the ISR. As exemplified by the PROTAC degrader PT-129, which targets the core stress granule proteins G3BP1/2 for degradation, it is possible to achieve profound and sustained disassembly of stress granules. This strategy disrupts the intercellular transfer of pro-survival mediators like ATF4 and overcomes resistance mechanisms rooted in stress adaptation (Dong et al., 2025). Additionally, nanobody-based therapeutics, characterized by their small size, superior tissue penetrability, and high affinity, present a promising avenue for targeting ISR-related signaling molecules located on cell membranes or within the complex TME.

### 4.2 Repurposed agents modulating ISR

Several clinically approved drugs exert modulatory effects on ISR signaling and could be repositioned to complement immunotherapy.

Metformin counteracts trained immunity by suppressing the Akt-mTOR-HIF-1α axis that governs glycolytic reprogramming in myeloid cells (Cheng et al., 2014). By doing so, it indirectly opposes immunometabolic changes linked to ISR activation. Ongoing clinical trials are investigating metformin in combination with ICIs to alleviate immunosuppression and improve outcomes in various solid tumors.

Reactive oxygen species (ROS), which accumulate due to mechanisms like TMED4 loss leading to IRE1α instability and impaired NRF2 function, can destabilize Foxp3 and compromise Treg suppressive capacity (Jiang et al., 2024). However, the antioxidant N-Acetylcysteine (NAC) can also scavenge ROS induced by certain chemotherapies, thereby inhibiting the release of oxidized mtDNA and subsequent activation of the cGAS/STING/type I interferon pathway. This action can paradoxically dampen the immunogenic response and reduce the efficacy of PD-L1 blockade (Qiao et al., 2023).

The potential of metformin in breast cancer immunotherapy requires further clarification. It can promote the endoplasmic reticulum-associated degradation of PD-L1 via AMPK-mediated phosphorylation, thereby enhancing T cell-mediated tumor killing, as demonstrated in humanized mouse models (Cha et al., 2018). A significant challenge is the absence of validated biomarkers for patient selection. The mechanism of NAC is multifaceted; for example, NF1 deficiency in breast cancer drives resistance to the PI3Kα inhibitor alpelisib by enhancing glycolysis and reducing ROS levels, a vulnerability that can be targeted by adding NAC to the treatment regimen (Auf der Maur et al., 2023). This supports the exploration of NAC as an adjunct to cell-based immunotherapies.

### 4.3 Biomarker-driven strategies

The successful clinical translation of ISR-targeting agents hinges on the development of robust predictive biomarkers to guide patient stratification and optimize therapeutic efficacy. This process follows a logical pipeline from biomarker discovery to clinical application.

The initial step involves identifying molecular features indicative of ISR activation. Transcriptomic signatures comprising key ISR-associated genes (ATF4, CHOP, GDF15) have been correlated with an immunosuppressive TME and poor response to ICIs in retrospective analyses. At the protein level, immunohistochemical (IHC) detection of p-eIF2α provides a direct and quantifiable histopathological measure of pathway activation in tumor biopsies and is under investigation as a companion diagnostic in early-phase trials (Guo et al., 2017).

Beyond tissue-based assays, liquid biopsies offer a non-invasive means to dynamically monitor ISR activation. Exosomes released by stressed tumor and immune cells carry specific proteins and RNAs that may serve as surrogate signatures of pathway activity. For instance, chronic ATF4 induction in CD8^+^ tumor-infiltrating lymphocytes (TILs) has been identified as a prognostic biomarker for T cell exhaustion and ICI resistance, highlighting its potential for treatment monitoring and early detection of resistance (Alicea Pauneto et al., 2025). The application of single-cell and spatial transcriptomics further refines this understanding by mapping ISR activation to specific cell populations and histological regions within the TME, enabling the development of AI-powered algorithms to predict ISR status from routine pathology slides.

The ultimate goal is to integrate these validated biomarkers into clinical decision-making. A high ISR gene signature or elevated p-eIF2α IHC staining could identify patients most likely to benefit from ISR-pathway inhibitors, either as monotherapy or in rational combinations with ICIs. This biomarker-driven strategy ensures that interventions are directed towards a patient population with a biologically defined vulnerability, thereby increasing the probability of clinical success and advancing the paradigm of precision oncology. A summary of current therapeutic agents, their mechanisms of action, and associated biomarker strategies for targeting the ISR is provided in Table 2.

**TABLE 2:** Clinical translation strategies for ISR targeting.

Approach	Agents	MoA	Combination partners	Stage/Biomarkers
Kinase inhibition	AMG-44 (PERKi)	Selective PERK blockade	Anti-PD-1/PD-L1	Phase I (p-eIF2α IHC)
	GCN2iB	GCN2 inactivation	Asparaginase/ICIs	Preclinical (high ASNS)
eIF2α signaling modulation	ISRIB	eIF2B complex activation	Radiotherapy/vaccines	Preclinical (low p-eIF2α)
Metabolic reprogramming	Metformin	Attenuates ISR signaling via mitochondrial complex I inhibition	ICIs (NCT04124433)	Phase II (HbA1c levels)
	N-acetylcysteine (NAC)	Scavenges ROS and relieves ER stress	RT/Immunotherapy	Preclinical (elevated TILs)
Biomarker strategy	ISR gene signature (ATF4/CHOP/GDF15)	Facilitates patient stratification	ISR inhibitors + ICIs	Retrospective validation

To translate ISR biology into testable clinical hypotheses, we outline a simple text-based workflow. Step 1: measure ISR state using orthogonal readouts such as p-eIF2α immunohistochemistry, ATF4-responsive transcriptional signatures, and “resolution-versus-escalation” context markers (GADD34/PPP1R15A versus CHOP/DDIT3), ideally considering temporal dynamics where longitudinal sampling is feasible. Step 2: co-stratify immune contexture using standard immunoprofiling to distinguish immune-inflamed versus immune-excluded/desert phenotypes and to capture dominant suppressive axes (myeloid-dominant suppression versus lymphoid-enriched states). Step 3: match mechanism-based combination prototypes. For example, ISR-high + myeloid-suppressive tumors may prioritize ISR modulation aligned with myeloid-reprogramming strategies to sensitize checkpoint blockade; ISR-high + immune-excluded tumors may emphasize regimens that promote trafficking and antigen presentation with carefully timed ISR modulation; whereas ISR-low + immune-inflamed tumors may be less suitable for broad ISR suppression and instead benefit from conventional ICI optimization. This framework is intended as a hypothesis-generating concept, not a prescriptive treatment algorithm, and it highlights where prospective biomarker validation is most needed.

### 4.4 Preclinical efficacy of combinatorial regimens

Preclinical studies have systematically explored the synergistic potential of combining ISR inhibitors with multiple therapeutic modalities. In TNBC mouse models, the PERK inhibitor AMG-44 in combination with paclitaxel markedly enhanced immunogenic cell death (Li X. et al., 2021), characterized by pronounced increases in calreticulin exposure and HMGB1 release, and promoted dendritic cell maturation, as reflected by elevated CD80/CD86 expression. This combination substantially improved the antitumor efficacy of chemotherapy and induced durable immune memory, as demonstrated by robust tumor rejection upon rechallenge.

In HER2^+^ breast cancer models, combining GCN2iB with radiotherapy reversed radiotherapy-induced, ATF4-dependent immunosuppression, markedly increased the balance of cytotoxic T cells over regulatory T cells, and significantly delayed tumor recurrence (Nakamura et al., 2018). Notably, the combination of ISRIB with dual PD-1/CTLA-4 blockade in metastatic breast cancer models overcame resistance to single-agent immune checkpoint inhibition, leading to a pronounced reduction in pulmonary metastatic burden (Wu et al., 2025). Collectively, these findings provide strong support for the clinical translation of ISR-targeted therapies in combination with chemotherapy, radiotherapy, and immunotherapy.

To move beyond enumerating combinations, we propose three pragmatic prototypes for integrating ISR modulation with immunotherapy in breast cancer: (1) myeloid reprogramming, aiming to reduce chronic stress programs that stabilize suppressive macrophage/MDSC states; (2) antigenicity and presentation enhancement, aligning ISR modulation with modalities that increase antigen release and cross-presentation; and (3) metabolic–stress normalization, mitigating nutrient/redox stress programs that enforce T-cell dysfunction and impaired effector fitness. Importantly, these strategies should explicitly consider a potential antagonism: overly suppressing stress signaling may diminish therapy-induced immunogenic cell death, antigen release, or innate danger signaling required for effective priming. Therefore, combination design should prioritize systematic testing of dose, timing, and sequencing, with pharmacodynamic readouts confirming relief of chronic, immune-suppressive ISR states while preserving stress-associated immunogenicity.

## 5 Challenges and future directions

While targeting ER stress and the ISR holds considerable promise for cancer therapy, several obstacles impede its clinical translation. Future investigations should prioritize resolving the following critical areas. Overcoming immunotherapy resistance is a critical goal in breast cancer management, which is further complicated by the need to optimize multimodal treatment strategies, including surgical approaches, as evidenced by studies evaluating long-term outcomes after local recurrence (Li Q. et al., 2021).

### 5.1 The hurdle of cell-type-specific targeting

The ISR exerts divergent, context-dependent effects: it promotes tumor survival and immune evasion in malignant cells, while in immune effectors like T cells and dendritic cells (DCs), it often leads to functional suppression and exhaustion. This duality necessitates the development of cell-selective modulation strategies. For instance, inhibiting PERK disrupts the UPR by blocking the PERK/ATF-4 axis; when combined with mRNA vaccines, this approach remodels the tumor microenvironment by increasing CD8^+^ T cell infiltration and promoting M1 macrophage polarization, thereby curbing melanoma progression and metastasis (Li et al., 2025).

Emerging platforms are exploring innovative solutions for precise targeting. The delivery of EGFR and PD-L1 siRNAs via iRGD-targeted solid lipid nanoparticles is enhanced by low-dose radiation, effectively inhibiting glioblastoma growth and extending survival (Erel-Akbaba et al., 2019). Alternatively, the ISR regulates NKG2D ligand expression, and novel anti-NKG2D antibodies can be deployed to modulate this immune axis for either blocking or enhancing tumor cell killing (Raynaud et al., 2020). Furthermore, suppression of the ISR, mediated by miR-183 downregulation and subsequent eIF2Bδ upregulation, facilitates metastasis in breast cancer stem cells (Gupta et al., 2023). Striking this balance is paramount, as systemic ISR inhibition could unintentionally eradicate cancer stem cells (CSCs) while potentially compromising the immune cells essential for tumor eradication.

### 5.2 Complexity of drug resistance mechanisms

The ISR constitutes a dynamic network replete with feedback regulation. For example, activation of the eIF2α-ATF4(GADD34)-CHOP axis during combined arginine deprivation and canavanine treatment induces profound ER stress and apoptosis, enabling radiosensitization in head and neck squamous cell carcinoma (Chen et al., 2021). Although CHOP-based immunochemotherapy achieves cures in over 60% of patients with large B-cell lymphoma, refractory cases typically face dismal outcomes (Sehn and Salles, 2021). These observations suggest that monotherapy targeting a single ISR node may yield limited durability, underscoring the need for rational combinations that concurrently target multiple pathway components or downstream effectors.

The ATF4–GADD34-CHOP network forms a regulatory circuit with intrinsic feedback loops that dynamically calibrate the cell’s fate decision under ER stress (Marton et al., 2022). This circuitry enables transient pathway activation, allowing tumor cells to survive the initial therapeutic insult and subsequently rewire their signaling networks. Adding another layer of complexity, epigenetic mechanisms fine-tune the ISR; for instance, METTL16-mediated m6A methylation stabilizes ATF4 mRNA, suppressing ferroptosis and driving cholangiocarcinoma progression (Zhao et al., 2025). This highlights the imperative for combination strategies that co-target the upstream kinases and the downstream epigenetic or transcriptional apparatus.

### 5.3 Optimization of combination therapies

Preclinical evidence indicates that combining ISR inhibitors with immune checkpoint blockers (anti-PD-1), chemotherapy, radiotherapy, or metabolic agents (L-asparaginase) can substantially augment anti-tumor efficacy. For instance, GCN2 inhibition alone fails to counteract GSK3α-mediated proteasomal adaptation, a mechanism that drives asparaginase resistance in leukemia via cytoplasmic body formation (Hinze et al., 2022). Subsequent research must rigorously validate the synergistic potential and safety profiles of these combinatorial regimens in humanized immune mouse models and early-phase clinical trials.

Leveraging the ISR signal itself to engineer conditionally activated cell therapies represents another innovative direction. For instance, one can engineer CAR-T cells in which CAR expression is controlled by an ATF4-responsive promoter, which is selectively activated by the amino acid scarcity of the tumor microenvironment. This design spatially restricts CAR expression to the tumor site, improving safety and sustaining anti-tumor potency (Manchon et al., 2025). This design ensures that CAR-T cells become fully activated and express high levels of the CAR predominantly within the TME, which is rich in ISR-activating signals. Consequently, this strategy enhances tumor-targeting specificity while reducing “on-target, off-tumor” toxicity in healthy tissues, ultimately improving therapeutic safety and anti-tumor potency. This innovative approach has shown controlled anti-tumor activity in humanized mouse models (Manchon et al., 2025).

The combinatorial potential extends well beyond conventional modalities. The activation of ATF4 under amino acid scarcity can be leveraged to engineer a tumor microenvironment-responsive CAR expression system, thereby improving both the safety and anti-tumor potency of CAR-T cells (Manchon et al., 2025). Similarly, the synergistic interplay between coxsackievirus B5 and DNA damage repair inhibitors provokes irreversible ER stress via the PERK pathway, promoting cell death in non-small cell lung cancer (Cui B. et al., 2023). To efficiently navigate this complex combinatorial landscape, Artificial Intelligence (AI) platforms are being deployed. For example, AI-driven multi-omics analysis has revealed that gain-of-function STING mutations elicit a PERK-mediated ISR in monocytes, resulting in T cell senescence in the autoinflammatory disorder SAVI (de Cevins et al., 2023).

### 5.4 Development of novel delivery systems

To enhance tumor-specific delivery of ISR modulators and minimize systemic toxicity, advanced delivery platforms—including nanoparticles, exosomes, and antibody-drug conjugates (ADCs)—are under intensive investigation. For instance, intraperitoneal administration of an IRE1α inhibitor blocks the IRE1α-XBP1 axis, suppressing ceramide synthesis and the release of pro-inflammatory extracellular vesicles (EVs), ultimately alleviating liver inflammation and injury in diet-induced steatohepatitis (Dasgupta et al., 2020).

Sophisticated delivery systems are pivotal for maximizing therapeutic index. Engineered exosomes derived from mesenchymal stem cells can be loaded with agents like ISRIB or ATF4-targeting siRNAs and functionalized with targeting ligands for precise TME delivery, offering superior biocompatibility and biodistribution. Furthermore, the BH3 mimetic ABT-737 synergizes with *Pseudomonas* exotoxin-based ADCs by inducing ATF4-mediated ER stress, overcoming resistance mechanisms and triggering apoptosis (Traini et al., 2010).

### 5.5 Single-cell technologies and biomarker development

The application of single-cell transcriptomic (scRNA-seq) and proteomic (CITE-seq) technologies can delineate the ISR status across diverse cell populations within the tumor microenvironment, facilitating the identification of predictive biomarkers for treatment response. For example, scRNA-seq analysis has revealed that ATF4 deficiency in host fibroblasts disrupts the activation of perivascular cancer-associated fibroblasts (CAFs) and collagen production, impairing tumor angiogenesis and progression (Verginadis et al., 2022). Such biomarkers are instrumental for patient stratification in precision oncology.

Spatial transcriptomics is transforming our understanding by mapping ISR activation (ATF4, XBP1s) to discrete histological regions within the tumor. This technology has identified ATF4 signaling in endothelial cells as a critical regulator of ischemia recovery and macrophage polarization in peripheral artery disease (Turiel et al., 2025). Supervised machine learning models trained on these spatial and single-cell datasets are enabling the development of digital pathology algorithms capable of predicting ISR activity and patient prognosis directly from conventional H&E or IHC-stained slides.

In the future, the integration of multi-omics data—such as scRNA-seq, CITE-seq, spatial transcriptomics, and metabolomics—using computational biology approaches will enable more precise mapping of the ISR activation landscape in clinical specimens. Leveraging artificial intelligence and machine learning models will allow for the extraction of ISR pathway activation signatures from these complex datasets and the development of digital pathology algorithms. These algorithms could potentially predict ISR activity and patient prognosis directly from routine H&E-stained slides, thereby significantly propelling the precise clinical application of ISR-targeted therapies.

### 5.6 Development and clinical translation of novel ISR modulators

Although several ISR inhibitors (the PERK inhibitor AMG44, the GCN2 inhibitor GCN2iB, the HRI inhibitor AMI) have advanced to preclinical or early clinical stages, their long-term safety, tolerability, and potential resistance mechanisms warrant comprehensive evaluation. A key preclinical finding demonstrates that PERK inhibition with GSK2606414 exerts a senolytic effect in glioblastoma by preventing the reversal of therapy-induced senescence (TIS), thereby inducing apoptosis in recurrent cells and suppressing tumor regeneration to improve overall survival *in vivo* (Ketkar et al., 2024). The advent of new-generation, highly selective inhibitors like AMG44 bolsters optimism for successful clinical application.

Preclinical studies indicate that GZD824, a multi-kinase inhibitor, exerts anti-tumor activity in endometrial cancer by modulating the GCN2-ATF4 pathway, supporting its further investigation in clinical trials for GCN2 inhibition (Liu D. et al., 2025). A major focus remains the development of ISR inhibitors with optimized oral bioavailability and tunable blood-brain barrier penetration (particularly for targeting brain metastases). Concurrently, exploring under-investigated ISR components, such as the HRI kinase, may unlock novel therapeutic opportunities.

### 5.7 Future clinical translation and personalized strategies

Despite the promising prospects of ISR-targeted therapies, their clinical translation faces multiple challenges. First, the potential adverse effects of ISR inhibition warrant careful consideration. For example, PERK inhibition may induce pancreatic toxicity, and systemic blockade of the ISR could impair stress-adaptive responses in normal tissues. Therefore, the development of tumor-selective delivery strategies, such as nanobody, drug conjugates or exosome-based carriers, represents an important future direction (Erel-Akbaba et al., 2019).

Second, biomarker-driven patient stratification is critical. Ongoing clinical trials are evaluating the feasibility of phosphorylated eIF2α IHC scores (Guo et al., 2017), ISR-related gene signatures, and circulating exosomal ATF4 mRNA as predictive biomarkers (Alicea Pauneto et al., 2025). In addition, artificial intelligence–assisted multi-omics platforms may enable extraction of ISR activity features from routine histopathological sections, allowing noninvasive and dynamic monitoring of therapeutic responses.

**FIGURE 2:**
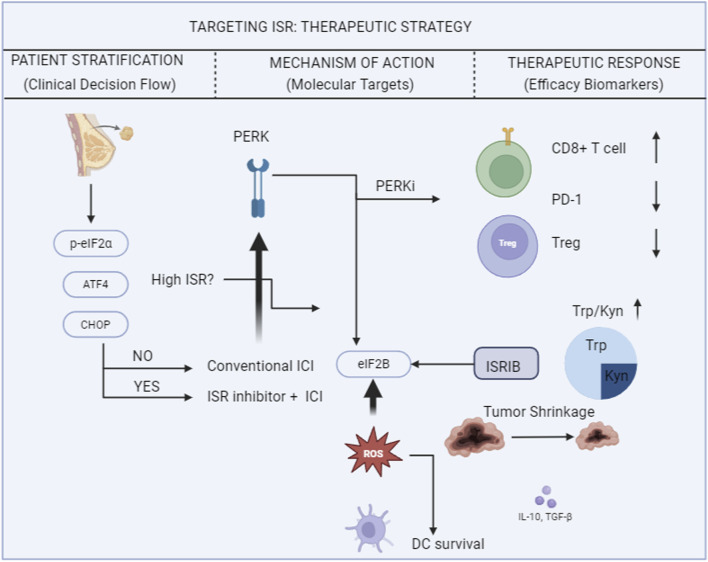
Roadmap of ISR-targeted therapeutic strategies. Created with BioRender.com. OB29GZF1RR.

Looking ahead, with the accumulation of clinical data, it is anticipated that a molecular classification framework based on ISR activity in breast cancer can be established. This will facilitate the design of personalized ISR-targeted combination strategies for different molecular subtypes, ultimately providing a means to overcome resistance to immunotherapy. A roadmap of ISR-targeted therapeutic strategies and translational considerations is presented in Figure 2.

Because the ISR core is a conserved homeostatic program, systemic targeting of PERK, GCN2, or downstream translation control (including ISRIB-like modulation) carries predictable on-target risks, including impaired stress tolerance in secretory tissues, altered metabolic adaptation, and unintended effects on immune-cell fitness during activation. To mitigate these liabilities while preserving anti-tumor benefit, several strategies deserve emphasis: (i) schedule optimization, such as intermittent dosing or short “priming” windows that avoid prolonged ISR suppression; (ii) tumor- or compartment-directed delivery, including targeted formulations where feasible; and (iii) rational sequencing with immunotherapies, timing ISR modulation to relieve chronic suppressive programs without blunting stress-associated immunogenicity required for effective priming. Across these strategies, incorporating pharmacodynamic monitoring (p-eIF2α/ATF4-responsive signatures and resolution markers such as GADD34/PPP1R15A versus CHOP/DDIT3) will be critical to define a therapeutic window and to guide patient selection.

## 6 Conclusion

This review synthesizes evidence establishing the ISR as a critical metabolic-immune interface and a fundamental mechanism of immunotherapy resistance in breast cancer. The ISR, orchestrated by upstream kinases (PERK, GCN2, PKR, HRI) and the central effector ATF4, enables tumor cells to adapt to metabolic stress while simultaneously orchestrating a broad immunosuppressive program within the tumor microenvironment. Key mechanisms include the upregulation of PD-L1, secretion of immunosuppressive exosomes, induction of metabolic competition, and direct impairment of T cell and dendritic cell function.

Targeting this axis represents a strategically viable approach to reverse immune evasion. Preclinical studies demonstrate compelling efficacy for various pharmacological strategies, including kinase-specific inhibitors, eIF2B activators like ISRIB, and repurposed agents such as metformin. These agents, particularly when combined with immune checkpoint blockade, chemotherapy, or radiotherapy, can resensitize tumors to immune-mediated attack.

However, the clinical translation of ISR-targeted therapies faces several challenges. The context-dependent and often opposing roles of the ISR in tumor versus immune cells necessitate the development of cell-selective targeting strategies. Furthermore, the pathway’s inherent feedback loops and redundancy demand rational combination therapies that co-target multiple nodes to achieve durable responses. The development and rigorous clinical validation of biomarkers, such as ISR gene signatures and p-eIF2α IHC, are paramount for effective patient stratification.

Future research must therefore prioritize the development of novel, highly selective ISR modulators, sophisticated delivery systems for spatial precision, and the integration of multi-omics data with artificial intelligence to guide personalized therapeutic sequencing. By systematically addressing these challenges, the strategic targeting of the ISR presents a promising frontier for overcoming immunotherapy resistance, offering new hope for patients with aggressive breast cancer subtypes.
